# Supplementary material for: Wireless battery free fully implantable multimodal recording and neuromodulation tools for songbirds
Source: Nat Commun. 2021 Mar 30;12:1968. doi: 10.1038/s41467-021-22138-8 (PMC8009877; doi:10.1038/s41467-021-22138-8)
Supplement: Supplementary file 1 — Supplementary Information [file 41467_2021_22138_MOESM1_ESM.pdf]

Supplementary Information

Wireless battery free fully implantable multimodal recording and neuromodulation tools for songbirds

Jokubas Ausra, Stephanie J. Munger, Amirhossein Azami, Alex Burton, Roberto Peralta, Julie E. Miller, Philipp Gutruf

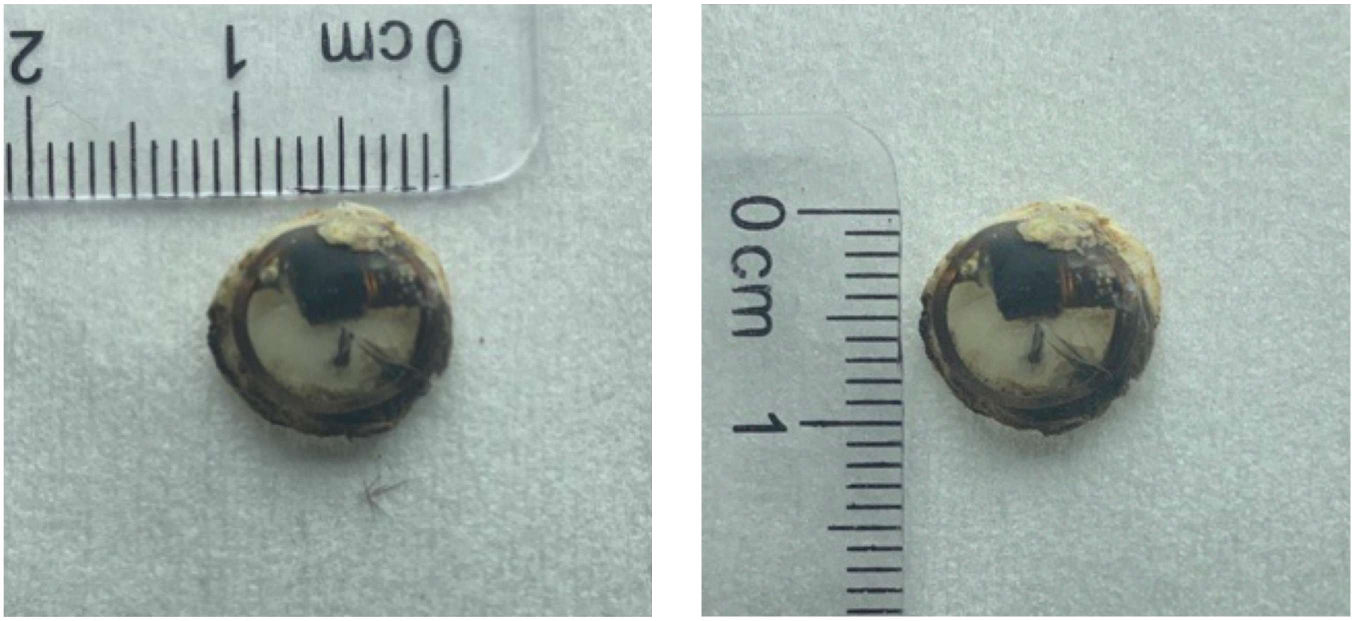

Figure S1. Photographic image of finch skull section with implanted device.

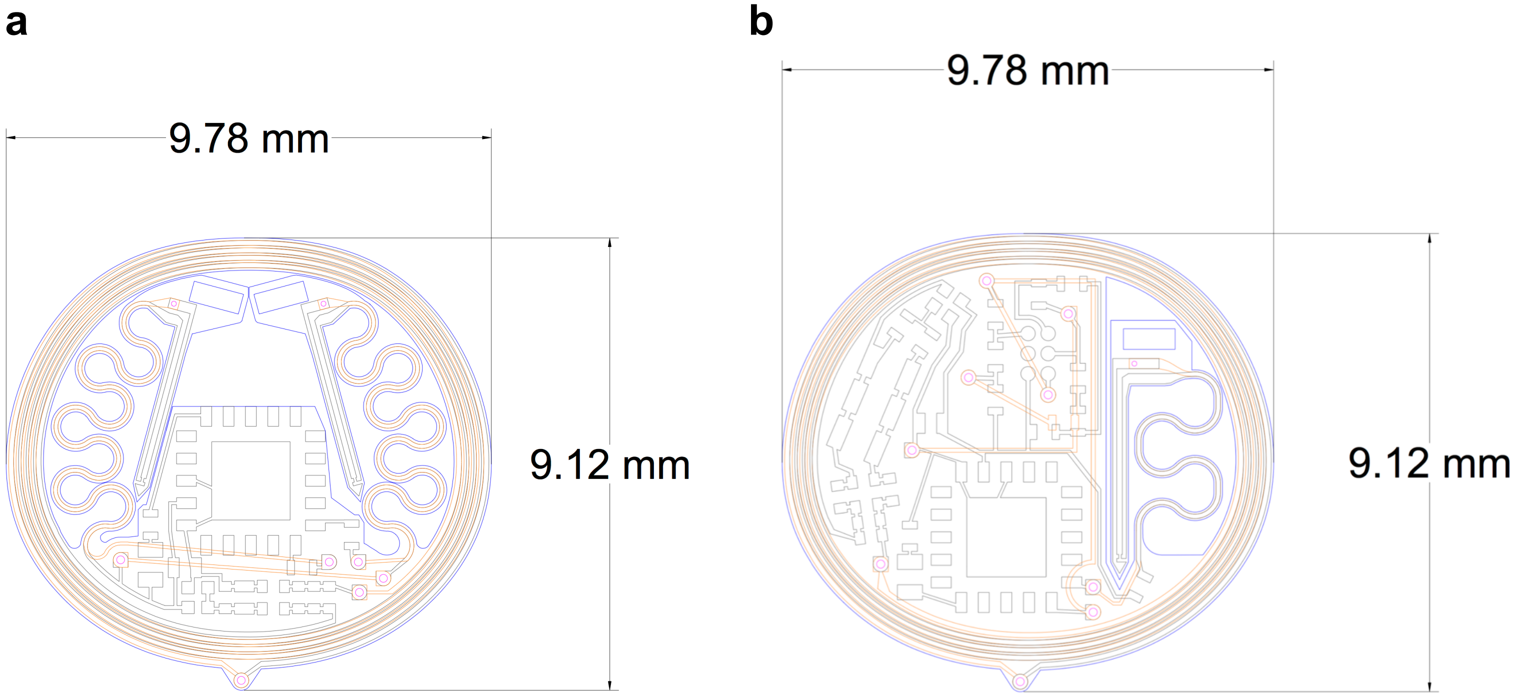

Figure S2. (a) Dual optogenetic probe device and (b) Multimodal optogenetic stimulator and thermography device with length and height indicated. Dual optogenetic probe device footprint occupies 50.62 mm^2. Multimodal optogenetic stimulator and thermography device footprint occupies 61.05 mm^2.

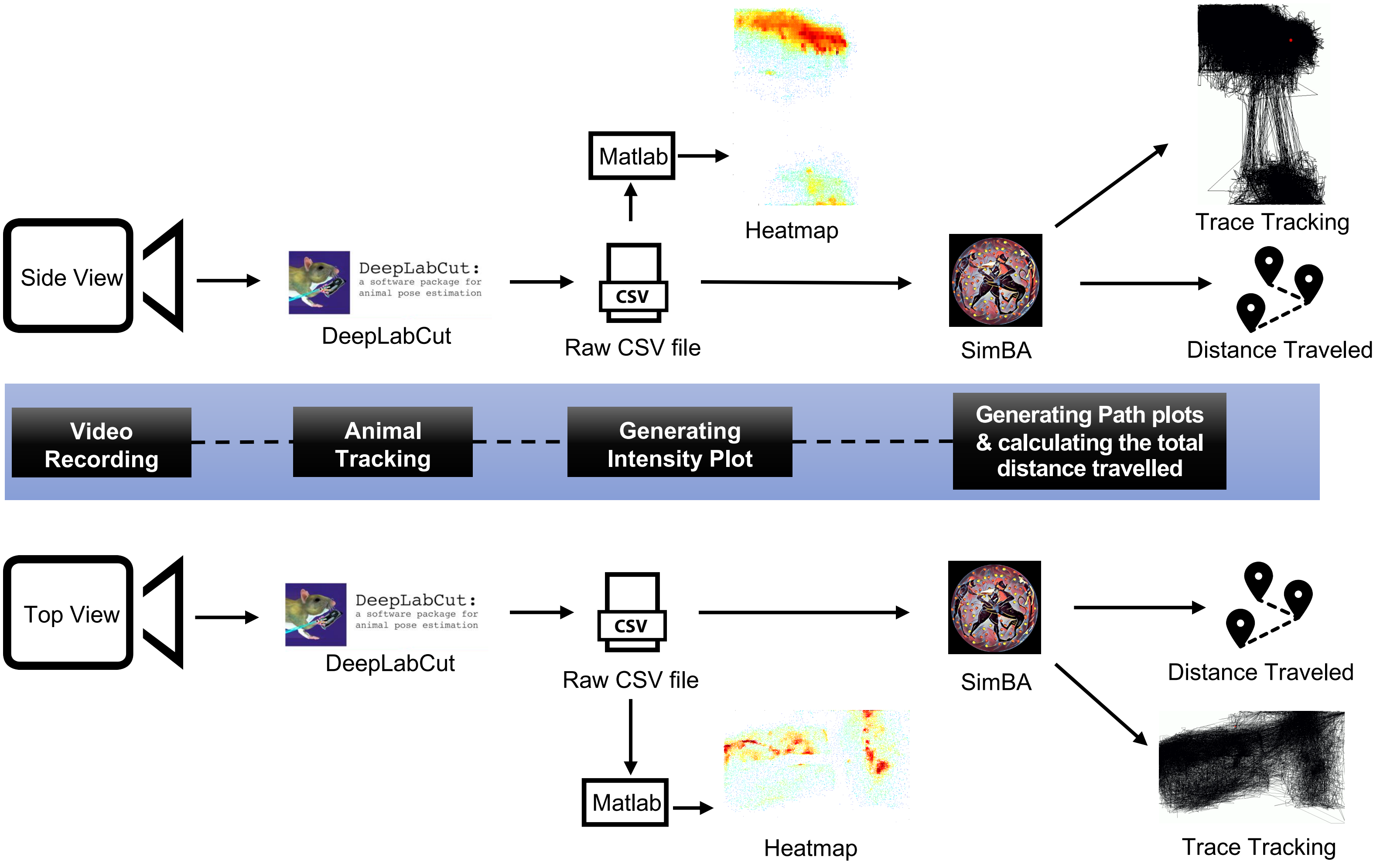

Figure S3. Flowchart of procedure for plotting motion tracking using deep learning software DeepLabCut and analysis with SimBA.

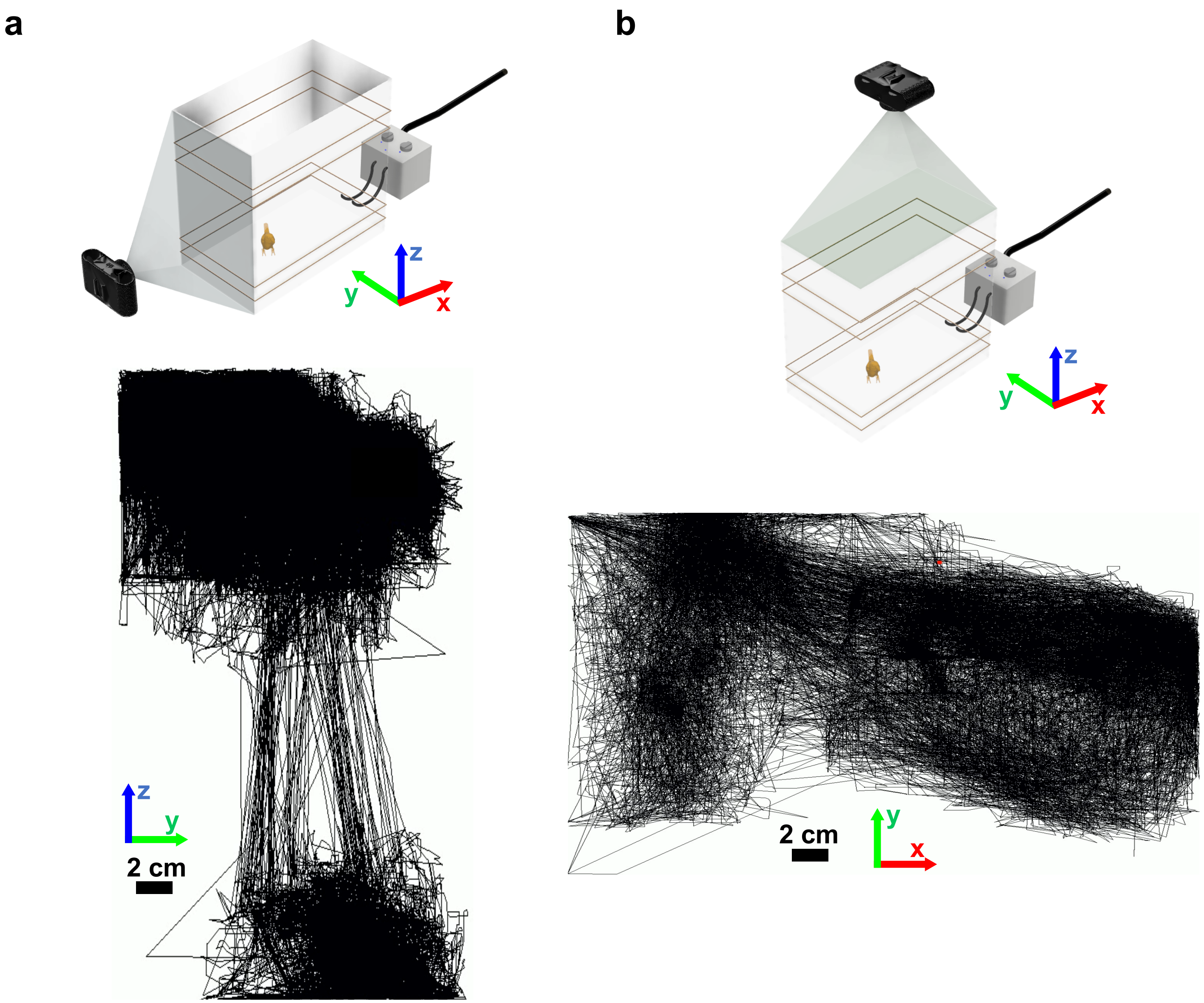

Figure S4. Trace tracking plots for the (a) side and (b) top view of the experimental arena created with SimBA software at a 99% confidence rate cutoff.

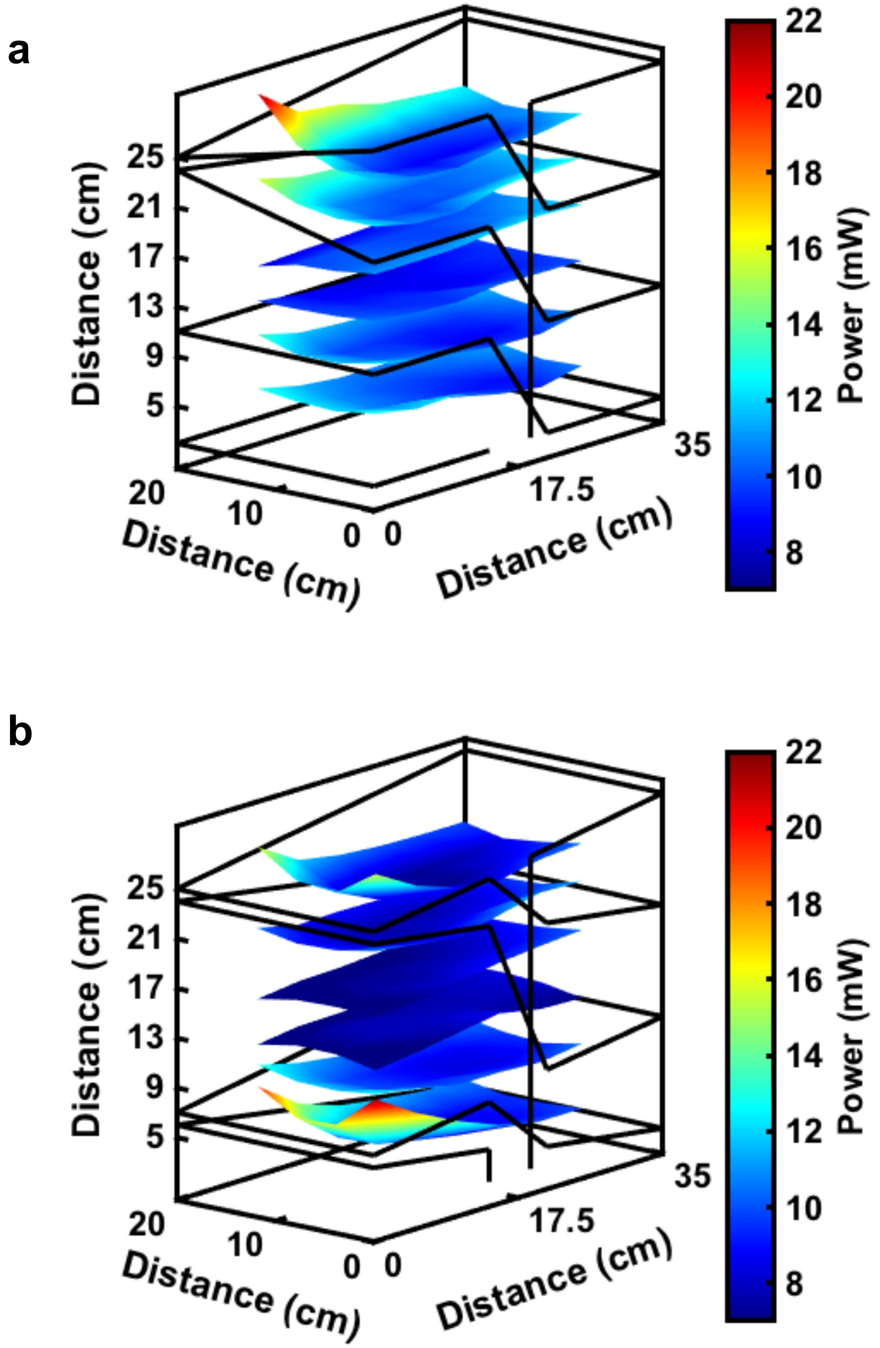

Figure S5. Power distribution inside a songbird arena. The antenna designs, conductive traces highlighted in black, (a) provides additional power to a corner of the cage at a height of 24.5 cm from the floor of the cage and (b) provides additional power to one side of the cage at a height of 6.5 cm and 24.5 cm from the floor of the cage.

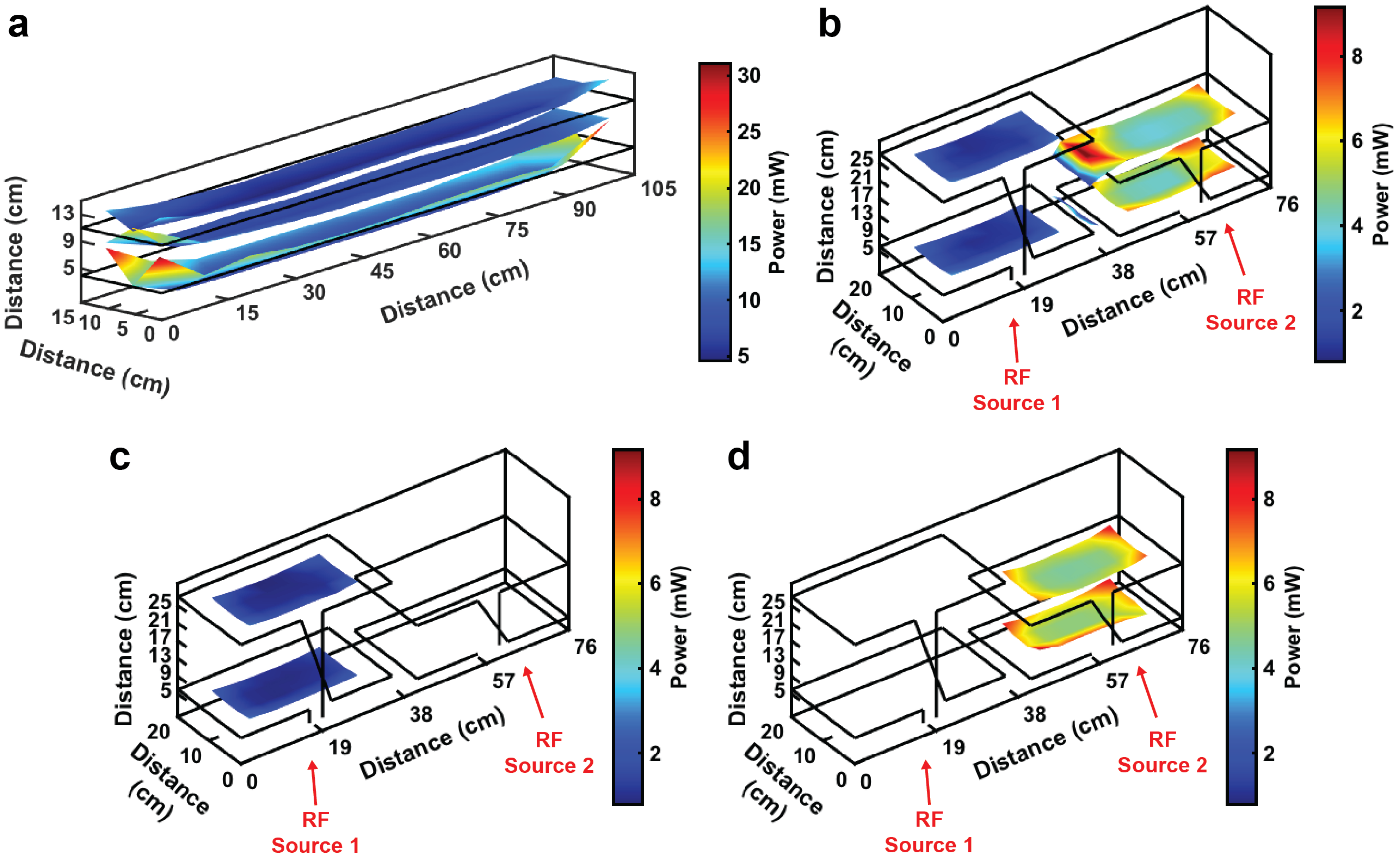

Figure S6. Alternative cage dimensions and sizes. (a) Power distribution at heights of 3, 7.5, and 12 cm from cage floor of a 15 x 15 x 105 cm cage with dual loop antenna and 10 W input power at heights of 4 and 11 cm from the cage floor. (b) Power distribution at heights 3, 6, 15, 27 cm from cage floor of a 20 x 70 x 35 cm cage composed of two antennas at heights of 3, 6, 15, 27 cm from the cage floor with 8 W power to both antennas on. The area in between the cages also receives RF power resulting in the ability to link multiple arenas to build large experimental spaces for freely flying animals. (c) Power distribution at heights 6 and 27 cm from cage floor of a 20 x 70 x 35 cm cage composed of two antennas at heights of 3, 6, 15, 27 cm from the cage floor with left antenna on at 8 W. (d) Power distribution at heights 3 and 15 cm from cage floor of a 20 x 70 x 35 cm cage composed of two antennas at heights of 3, 6, 15, 27 cm from the cage floor with right antenna on at 8 W.

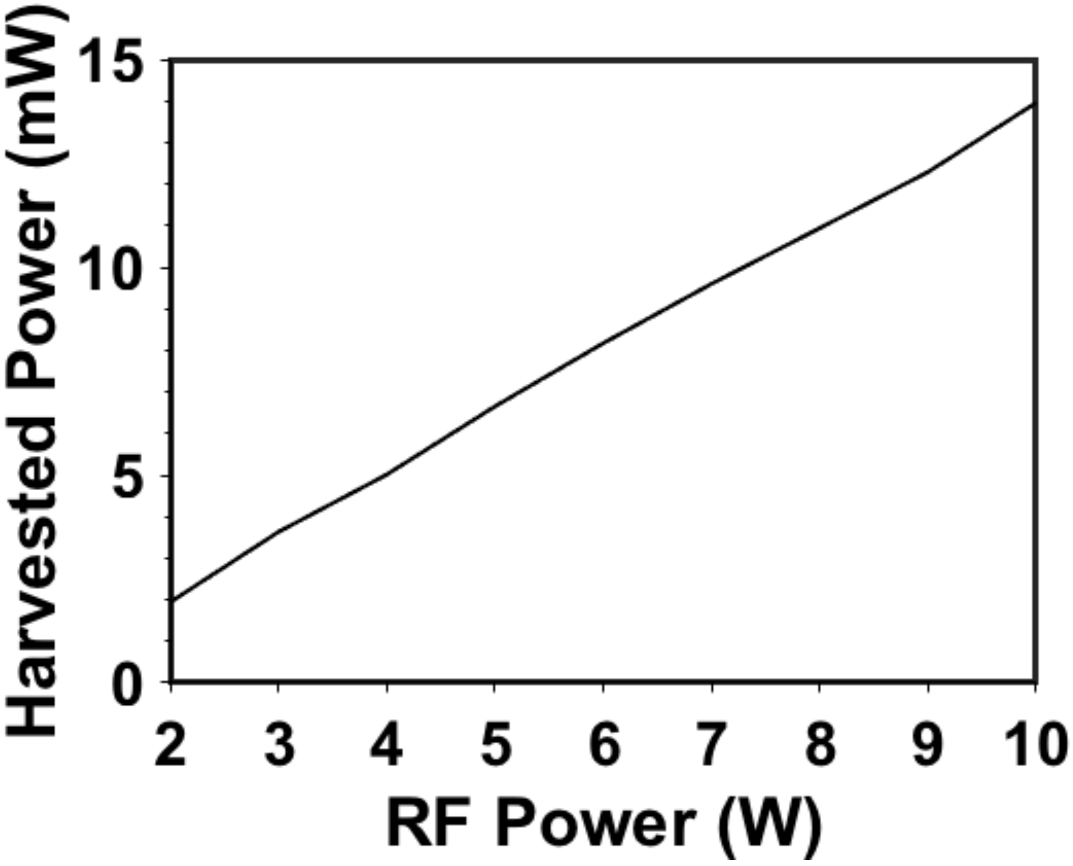

Figure S7. Input RF power dependent power harvesting in a 35 x 35 x 20 cm cage in the center at a height of 25 cm.

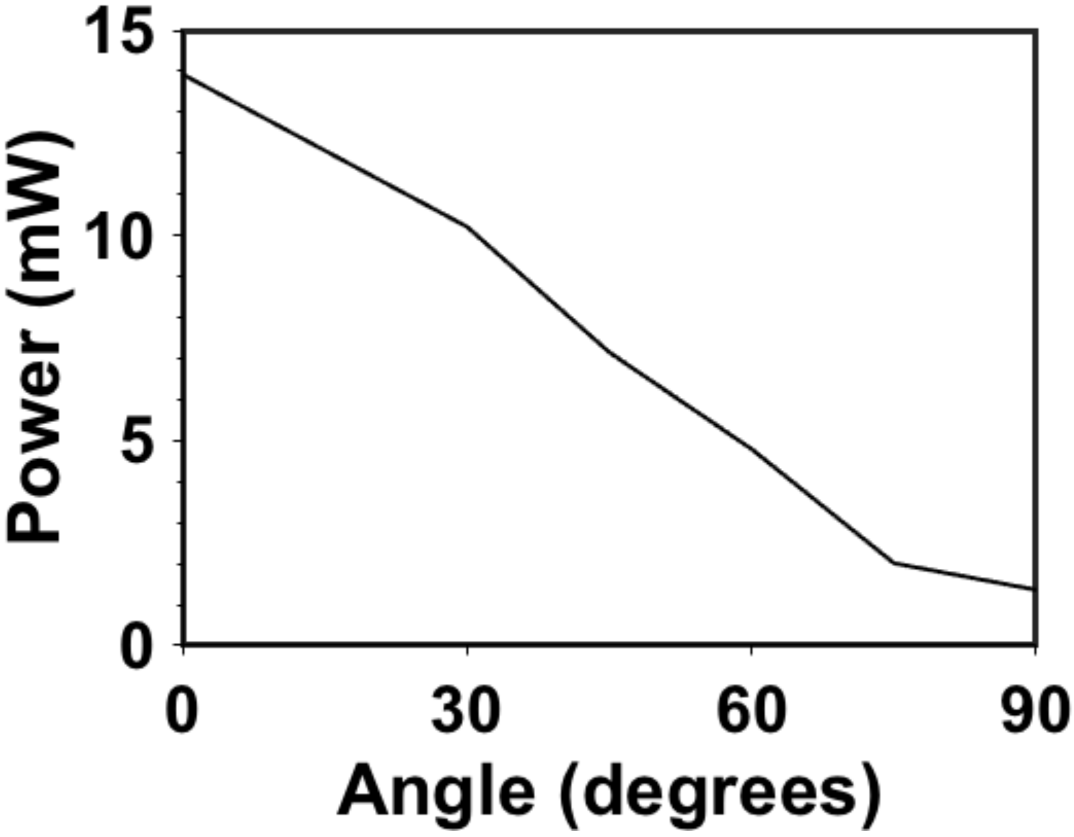

Figure S8. Angle dependent power harvesting in a 35 x 35 x 20 cm cage in the center at a height of 25 cm with an input power of 10 W.

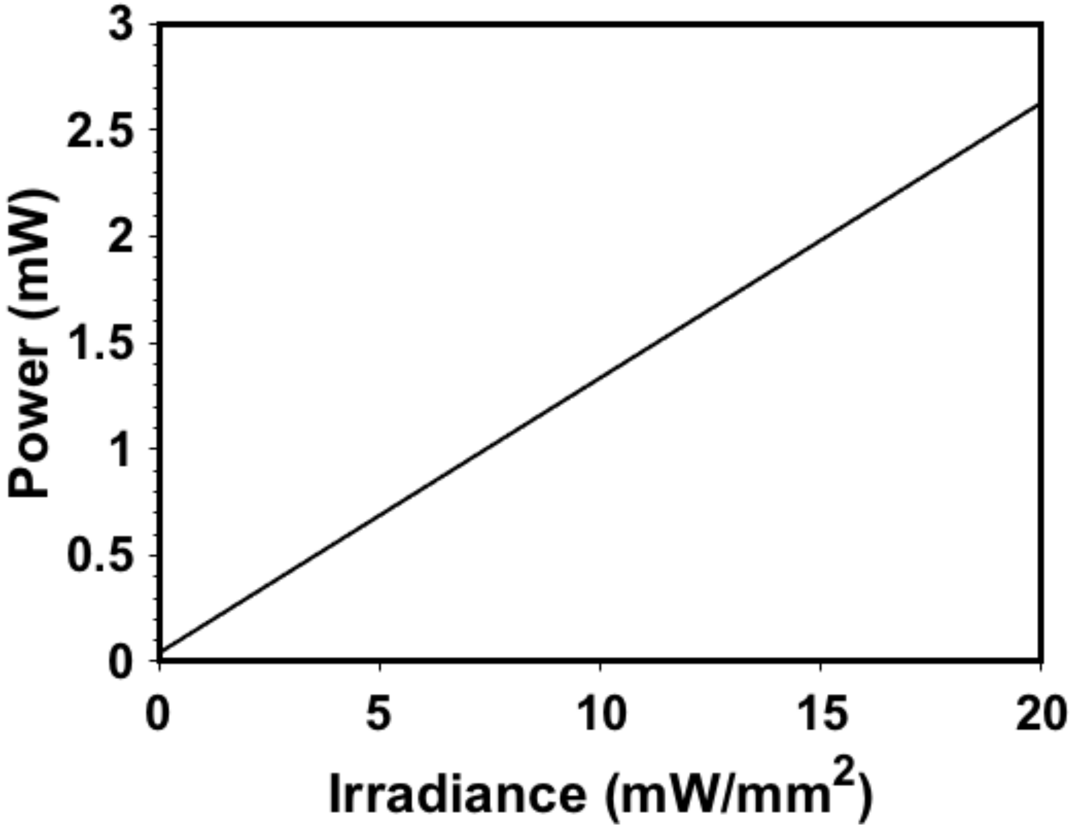

Figure S9. Graph of intensities and required average operational powers for each intensity (15% duty cycle).

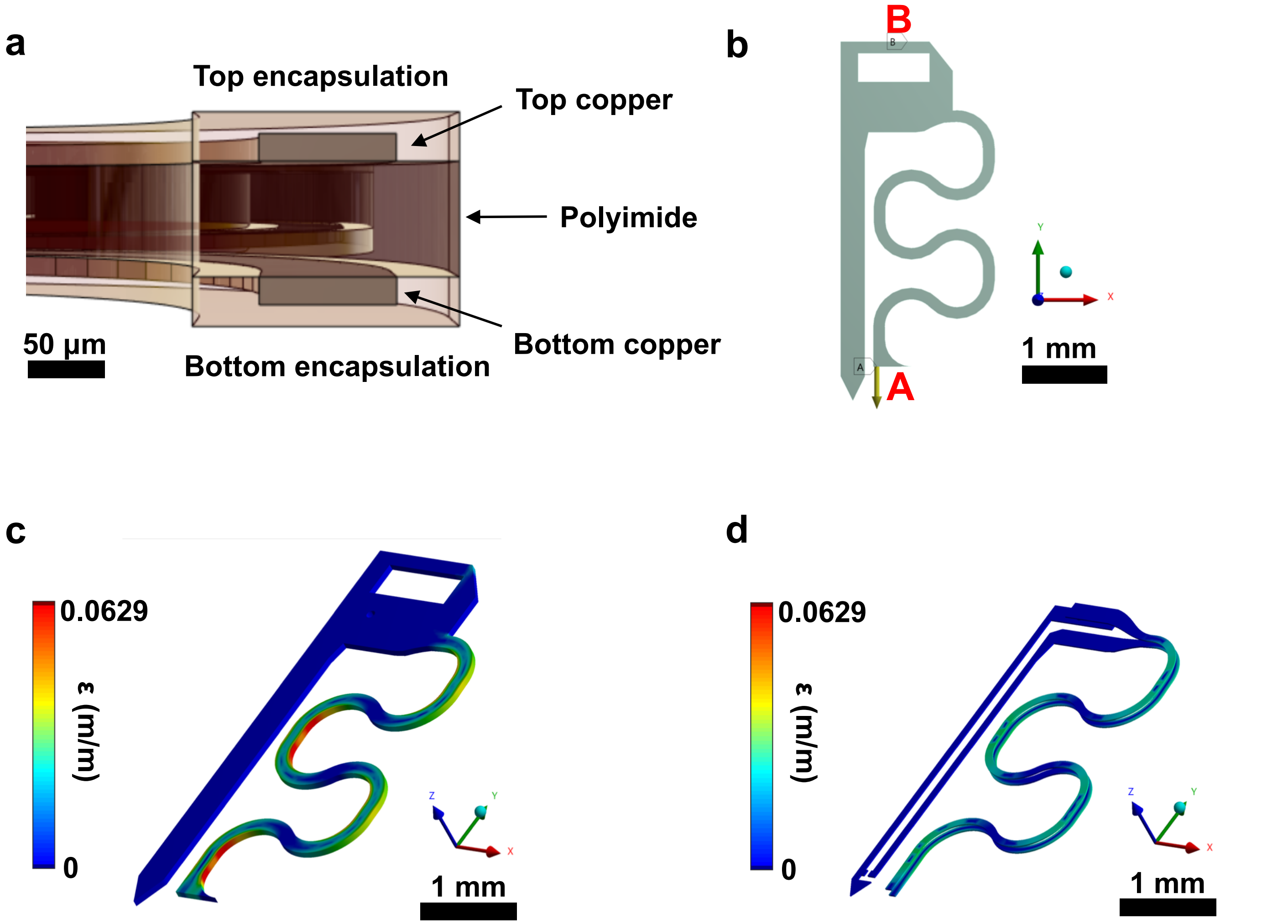

Figure S10. Mechanical characterization of Multimodal optogenetic stimulator and thermography device with serpentine at maximum displacement of 2.1 mm (55%) showing (a) cross-sectional schematic of probe layers, (b) direction of displacement indicated with arrow (point A) and location of fixed support (point B), (c) isometric view of serpentine with polyimide exposed, (d) isometric view of serpentine with copper exposed.

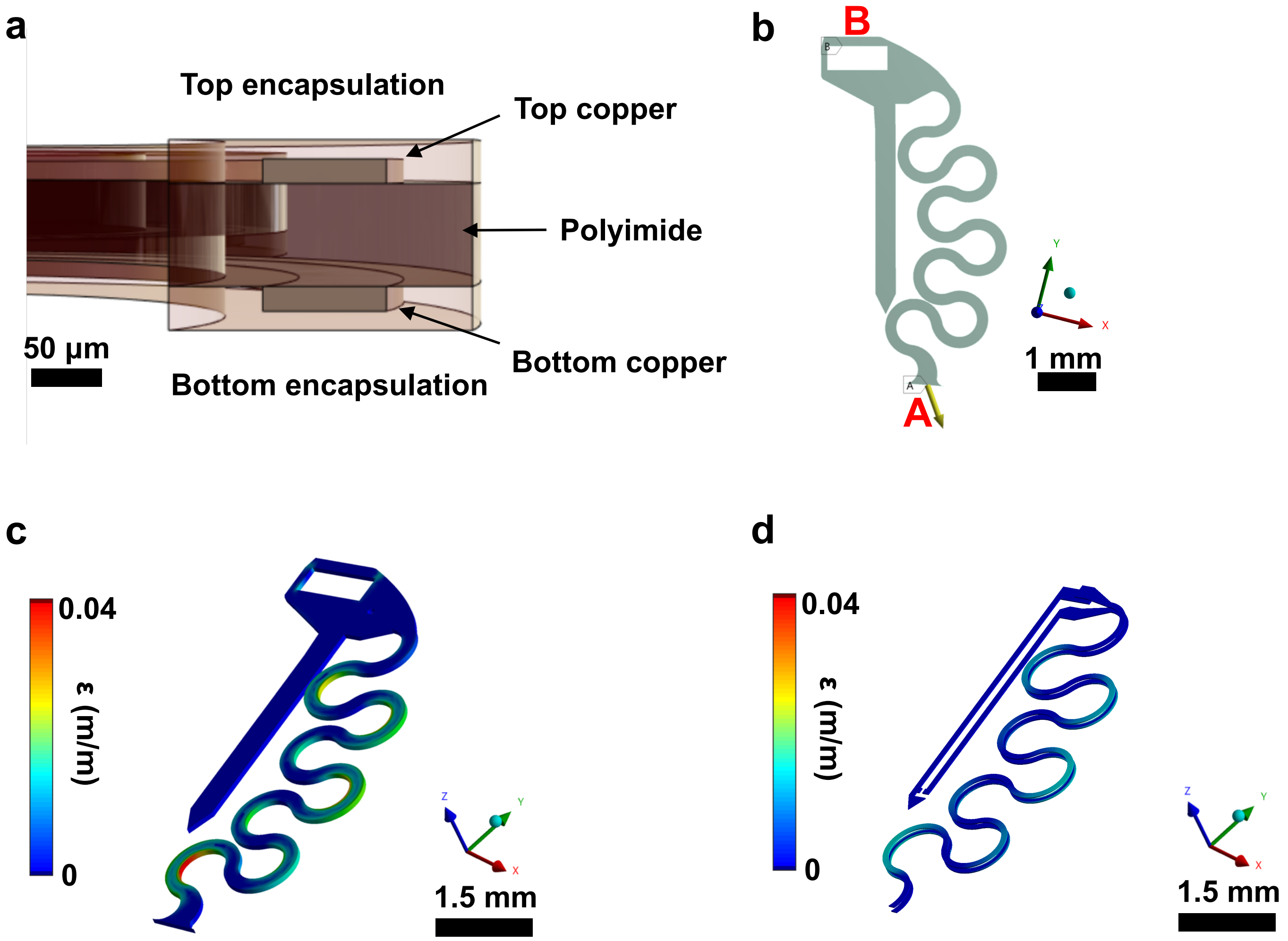

Figure S11. Mechanical characterization of dual optogenetic probe device with serpentine at maximum displacement of 1.2 mm (24%) showing (a) cross-sectional schematic of probe layers, (b) direction of displacement indicated with arrow (point A) and location of fixed support (point B), (c) isometric view of serpentine with polyimide exposed, (d) isometric view of serpentine with copper exposed.

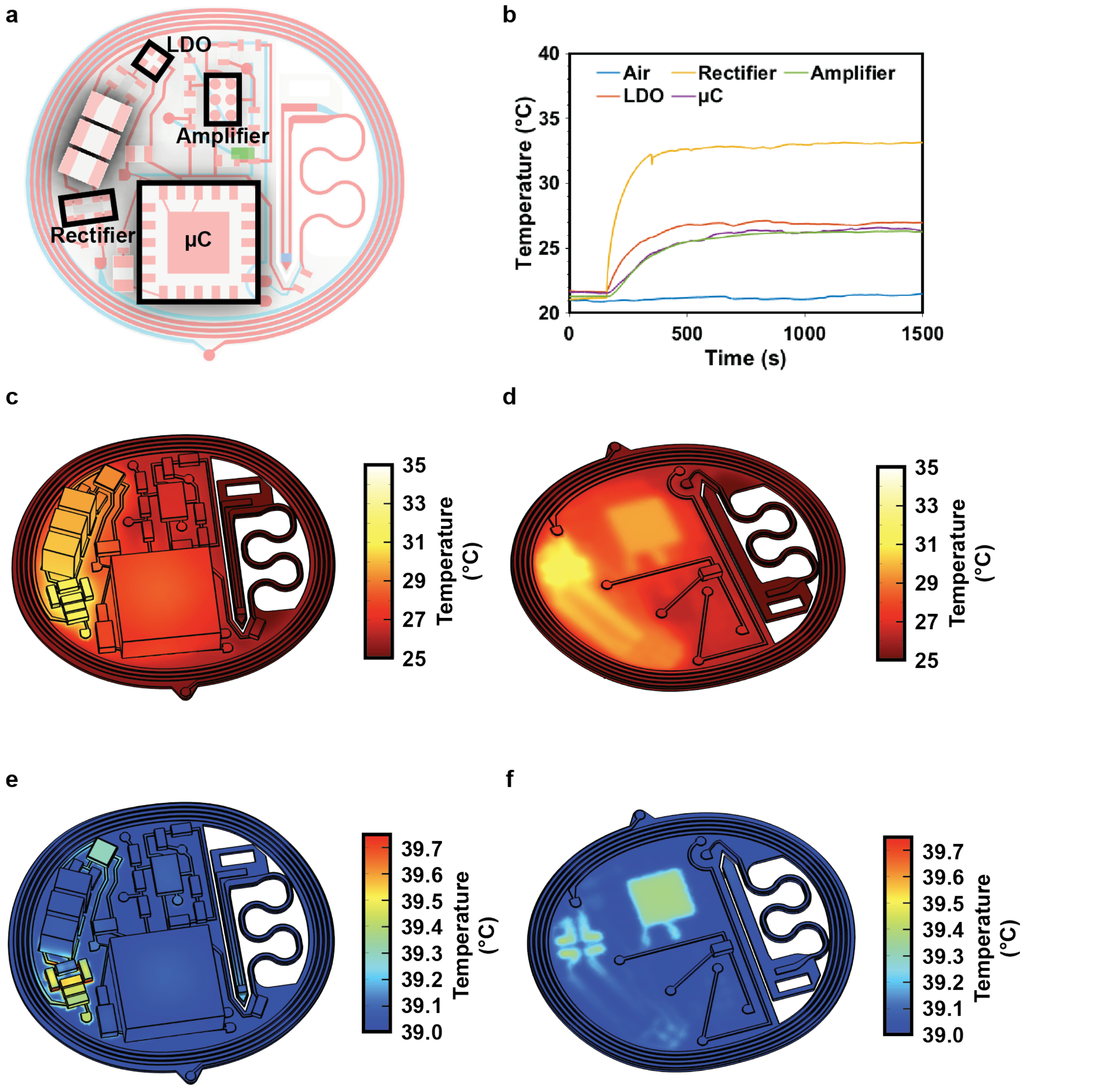

Figure S12. Steady state temperature measurements of device components during operation. (a) Schematic of device components indicating location of investigated components. (b) Steady state temperatures for LDO, rectifier, μC, and amplifier during operation (3W RF input into dual loop antenna 15 cm in diameter) in air. (c) Top view steady state thermal FEA analysis of device during operation in air without convection with initial temperature of 22 °C. Temperature increases by 9.57 °C for rectifier, 7.2 °C for LDO, 4.8 °C for amplifier, and 6.4 °C for μC. (d) Corresponding bottom view steady state thermal impact analysis. Temperature increases by 4.5 °C for NTC. (e) Top view steady state thermal impact analysis of device during operation in PBS with initial temperature of 39 °C. Temperature increases by 0.510 °C for rectifier, 0.301 °C for LDO, 0.085 °C for amplifier, and 0.085 °C for μC. (f) Corresponding bottom view steady state thermal impact analysis. Temperature increases at the NTC thermal sensor are 0.005 °C.

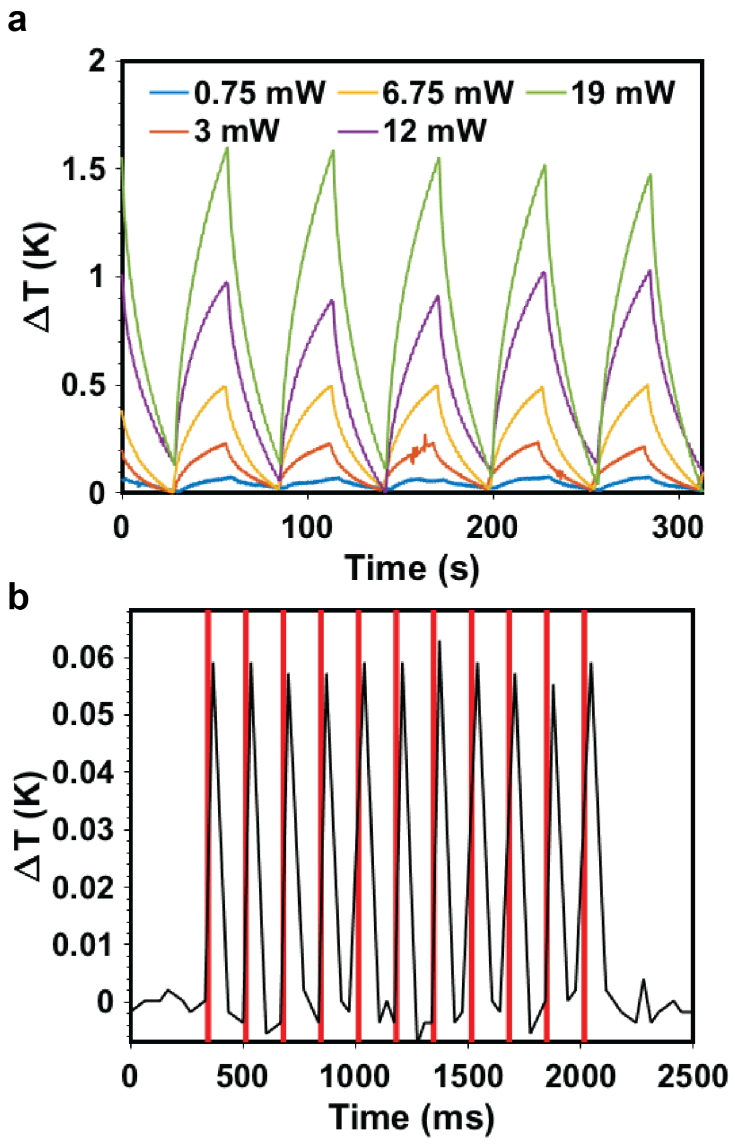

Figure S13. Graphs showing thermistor response to pulsed increase in temperature. (a) Temperature sensitivity at varying input powers of a heating coil placed in contact with the sensor and (b) temporal sensitivity of thermistor with heat applied at 6 Hz, 15% duty cycle, and 0.75 mW (red area).

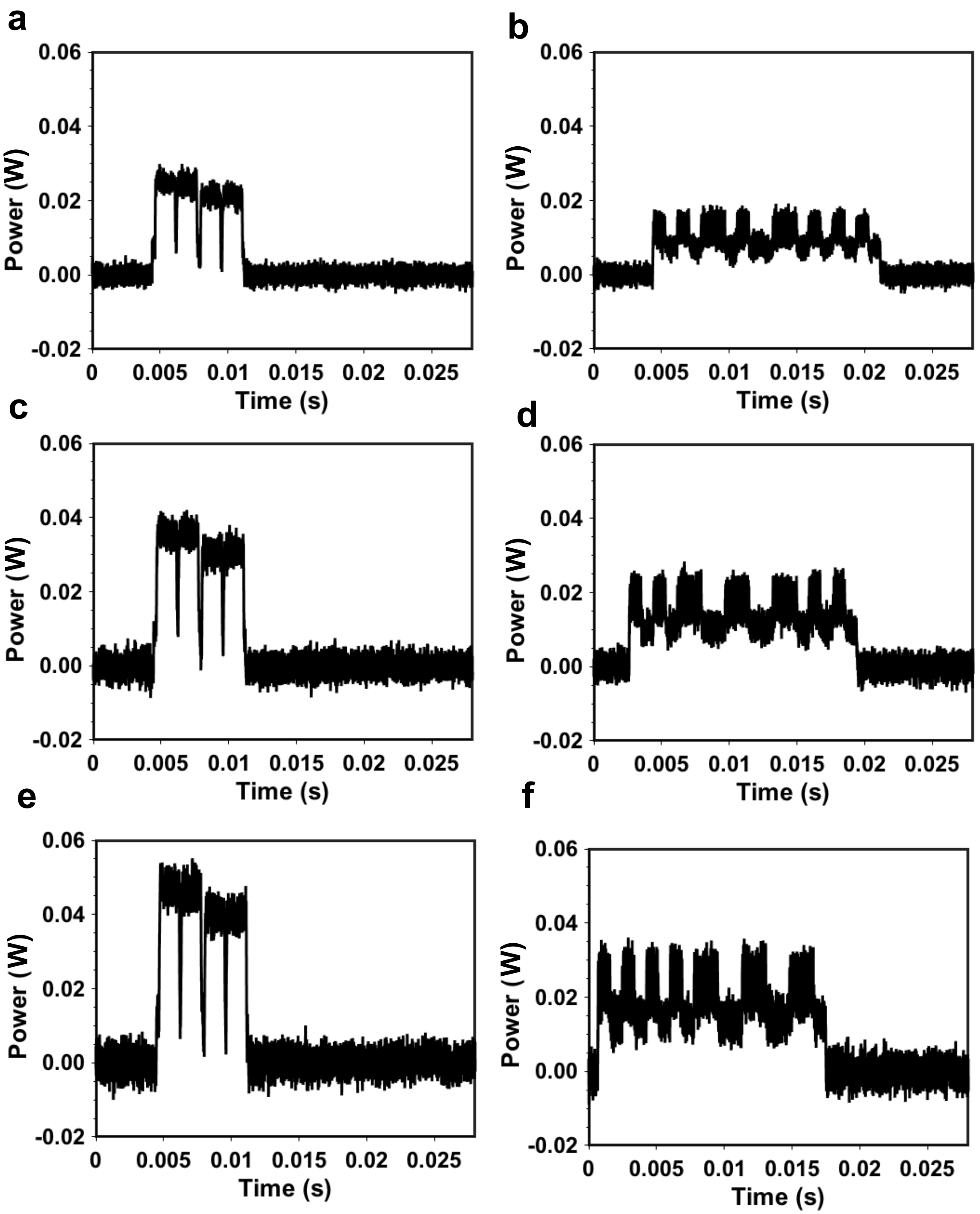

Figure S14. Power into the LDO of the Multimodal optogenetic stimulator and thermography device with input voltage of (a) 2.9 V during a digitalization event, (b) 2.9 V during a sending event, (c) 4.2 V during a digitalization event, (d) 4.2 V during a sending event, (e) 5.5 V during a digitalization event, (f) 5.5 V during a sending event.

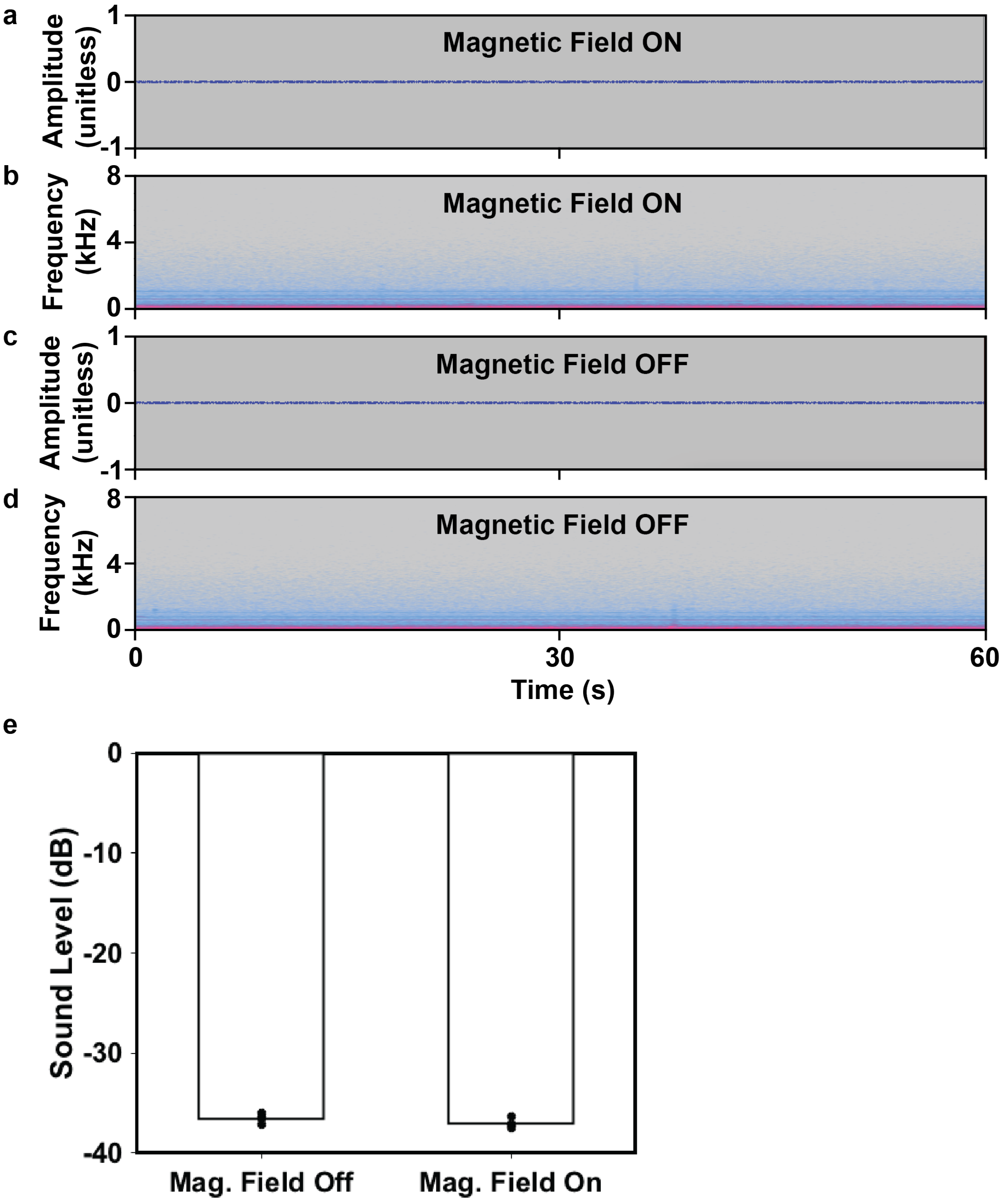

Figure S15. Noise characterization of sound chamber without animal occupation. (a) Amplitude of noise with magnetic field on at 10 W, (b) frequency analysis with magnetic field on at 10 W, (c) amplitude of noise with magnetic field off, (d) frequency analysis with magnetic field off. (e) Comparison of noise levels in chamber with magnetic field off and magnetic field on at 10 W presented as mean values +/- SEM.

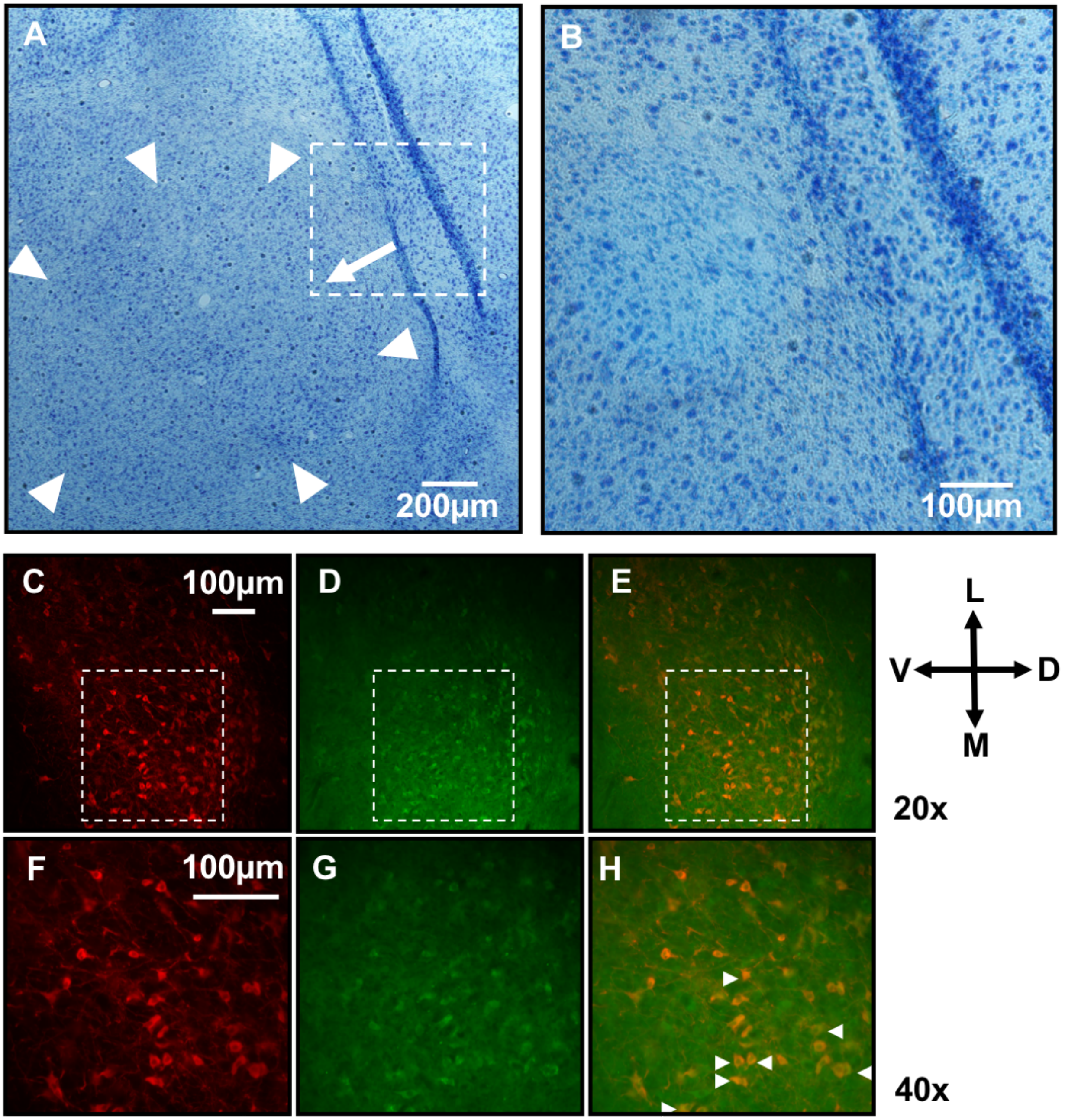

Figure S16. (a) Nissl staining in Area X-basal ganglia region. White arrows indicate spherical shape of Area X and the higher density of Nissl bodies within it. The µ-ILED probe track is shown with direction of light scatter (arrow). Histological assessment of probe placement in this region was conducted by sampling three slides (15 total tissue sections) from bird Subject C. (b) Inset from A shown at higher magnification. Probe diameter ~300µm. (c) Red signal denotes staining for tyrosine hydroxylase, the enzyme for dopamine biosynthesis, in coronal brain section three to four weeks after virus injection. Staining patterns were consistently repeated across multiple tissue sections per slide and three slides from this region were sampled for this. (d) Green signal denotes virally-driven Green Fluorescent Protein (GFP) expression. (e) Merged image of c-d. (f) Inset from c at higher magnification. (g) Inset from d at higher magnification. (h) Inset from e at higher magnification. Arrows indicate some examples of GFP signal in dopaminergic neurons. Coronal Section Orientation: D-dorsal, V-ventral, M-medial, L-lateral. See Methods: Tissue Histology.

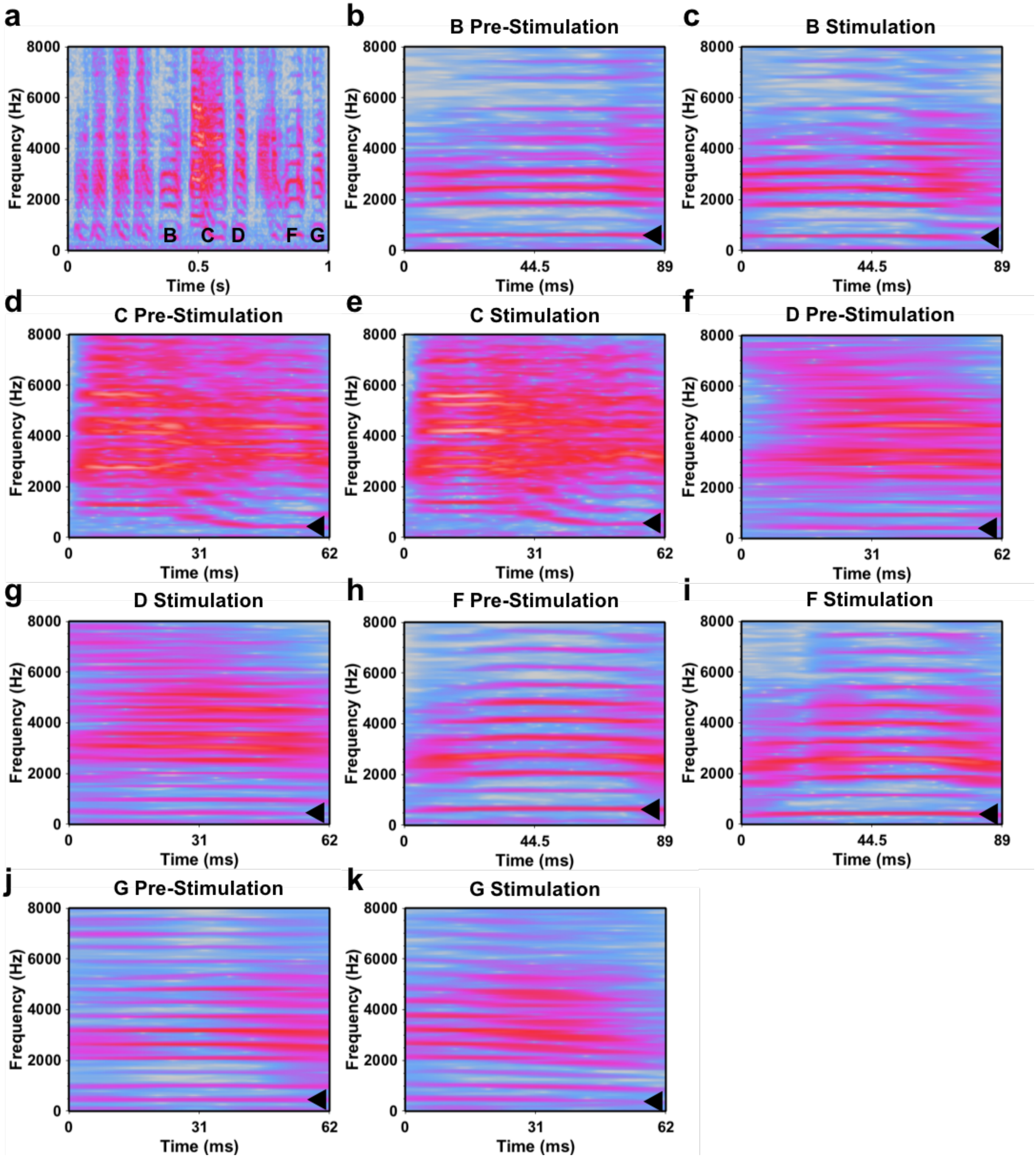

Figure S17. (a) Spectrogram of birdsong motif from finch Subject A and individual harmonic syllables analyzed with arrow denoting where f_o is measured for (b) B pre-stimulation, (c) B during stimulation, (d) C pre-stimulation, (e) C during stimulation; only the harmonic portion of this syllable was analyzed, (f) D pre-stimulation, (g) D during stimulation, (h) F pre-stimulation, (i) F during stimulation, (j) G pre-stimulation, (k) G during stimulation.

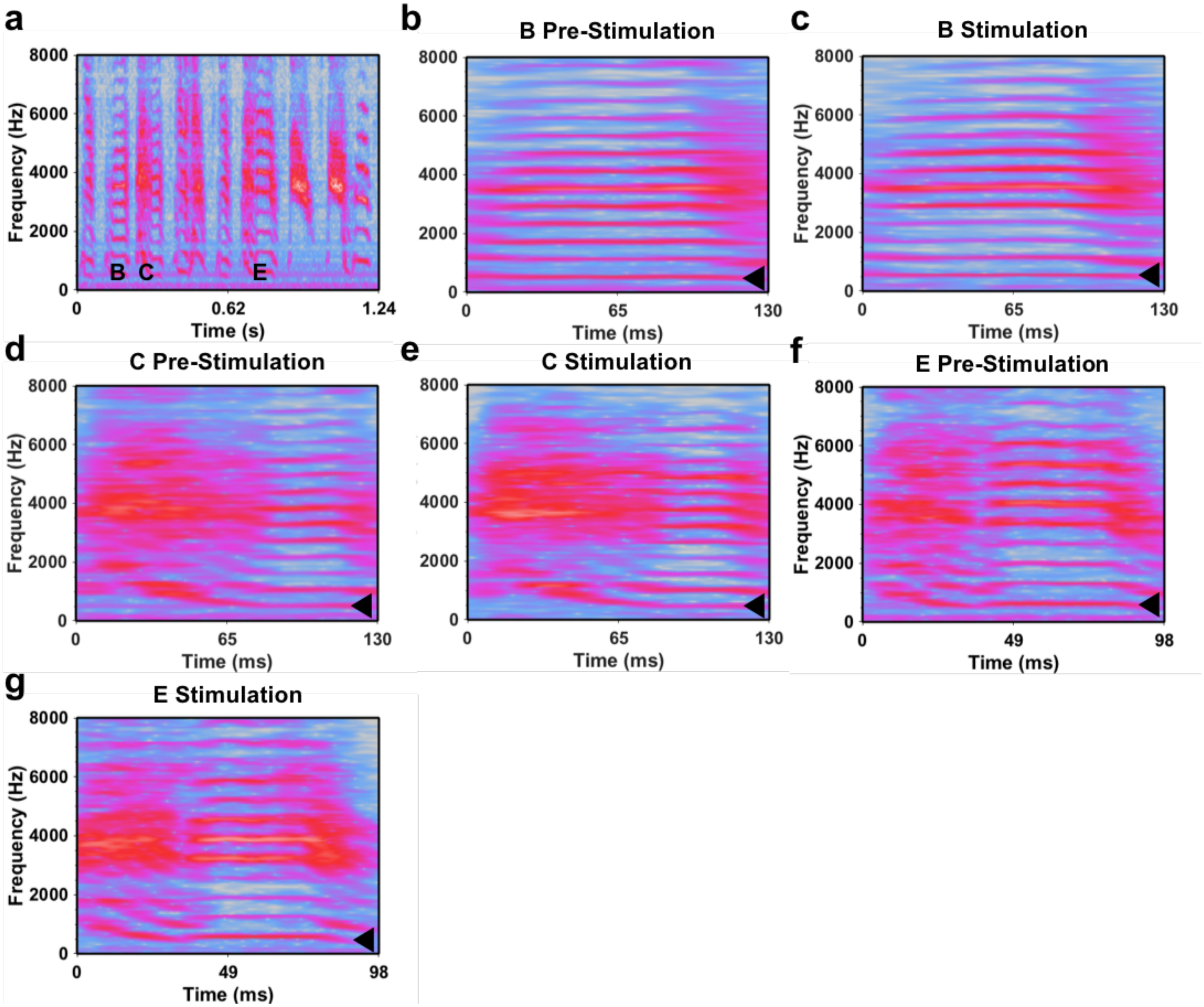

Figure S18. (a) Spectrogram of birdsong motif from finch Subject B and individual syllables harmonic portions analyzed with arrow denoting where f_o is measured for (b) B pre-stimulation, (c) B during stimulation, (d) C pre-stimulation, (e) C during stimulation, (f) E pre-stimulation, (g) during stimulation.

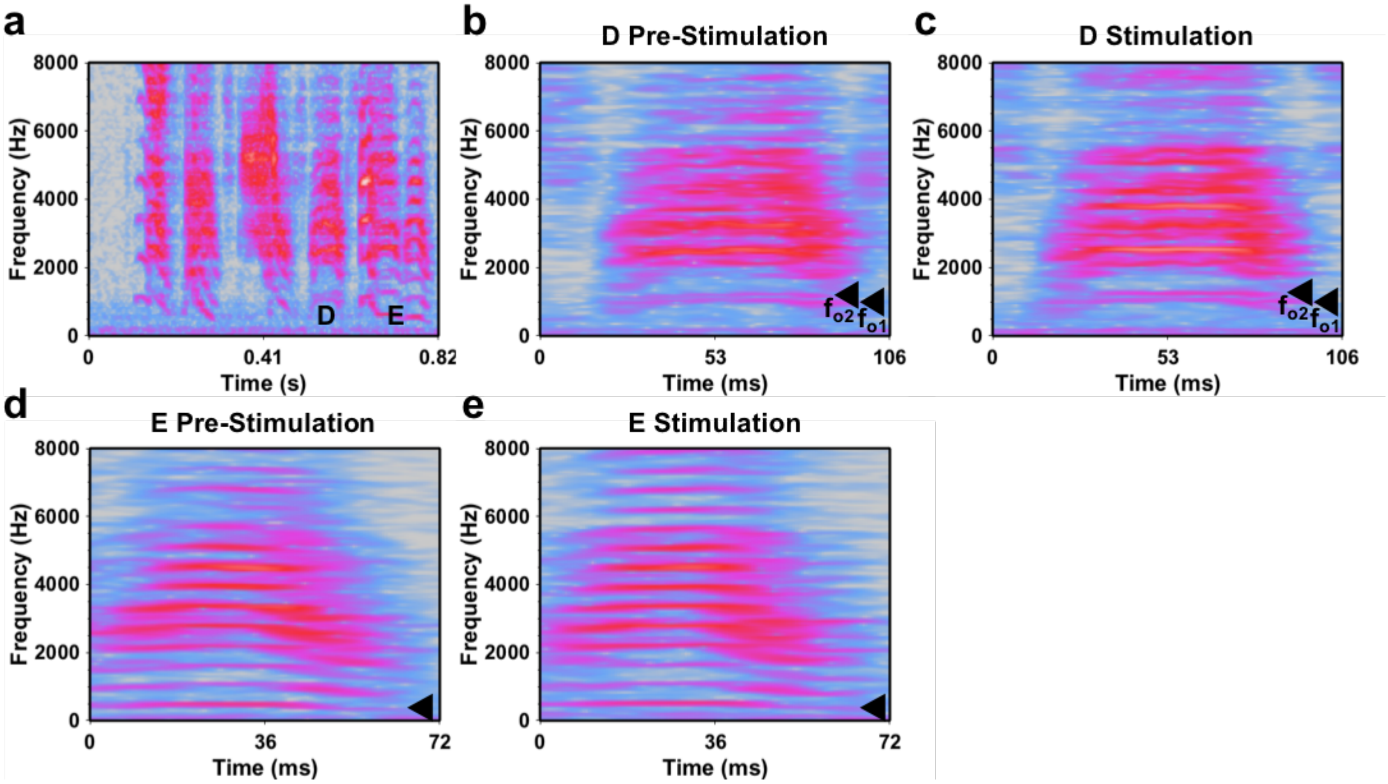

Figure S19. (a) Spectrogram of birdsong motif from finch Subject C and individual syllables with arrow denoting where f_o is measured for (b) D pre-stimulation, (c) D during stimulation, with two f_os denoted, (d) E pre-stimulation, (e) E during stimulation; only harmonic portion of E was analyzed.

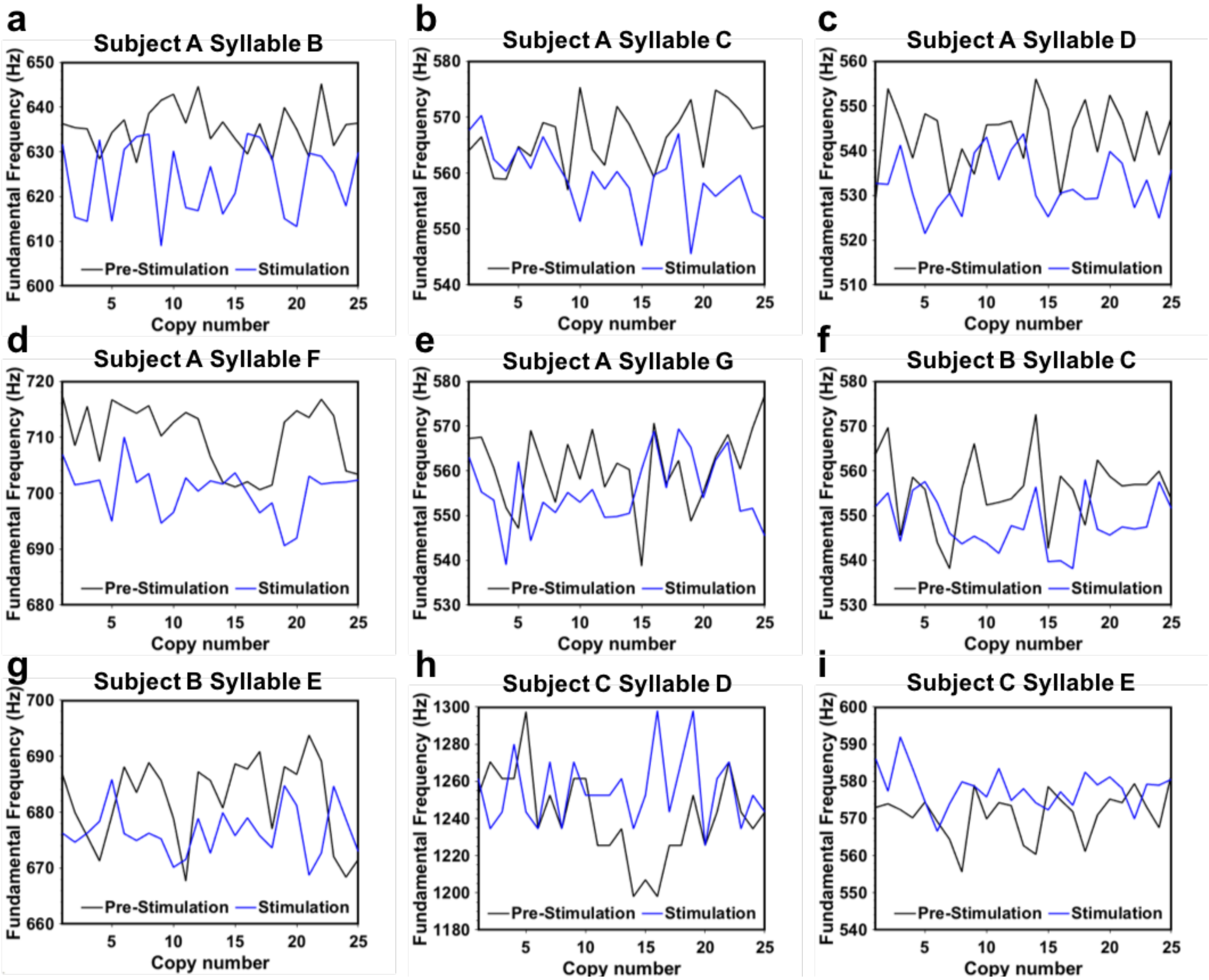

Figure S20. Fundamental frequency (f_o) scores of pre-stimulation (black line) and post stimulation (blue line) over 25 copies for (a) subject A (n = 1) and syllable B, (b) syllable C, (c) syllable D, (d) syllable F, (e) syllable G, (f) subject B (n = 1) and syllable C, (g) syllable E, (h) subject C (n = 1) and syllable D f_{o2}, (i) syllable E. Pre-stimulation versus stimulation comparisons for each plot were significant at $p<0.05$, Two-sided Wilcoxon signed rank; P-values are as follows: Subject A: Syllable B, p=0.000; Syllable C, p=0.001; Syllable D, p=0.000; Syllable F, p=0.000; Syllable G, p=0.017; Subject B: Syllable C, p=0.001; Syllable E, p=0.007; Subject C: Syllable D, f_{o2}, p=0.025; Syllable E, p=0.001; see Methods for statistical analyses.

Subject	Syllable	Pre-Stimulation		Stimulation	
		Mean	Standard Deviation	Mean	Standard Deviation
A	B, p<0.000	635.497	4.927	623.994	8.141
	C, p<0.001	566.439	5.262	559.031	6.048
	D, p<0.000	543.542	7.433	532.592	6.101
	F, p<0.000	710.094	5.881	700.524	4.355
	G, p<0.017	560.79	8.618	555.415	7.694
B	B, p<0.353	608.712	5.562	606.544	4.997
	C, p<0.001	555.849	8.051	548.342	5.974
	E, p<0.007	682.138	7.54	676.603	4.373
C	D, fo1, p<0.26	1080.28	20.776	1074.82	18.049
	D, fo2, p<0.025	1241.64	23.49	1255.08	19.303
	E, p<0.001	571.269	6.385	578.061	5.285

Table ST1. Table documenting mean pitch score and standard deviation values over 25 copies of either pre-stimulation or stimulation recordings. Two-sided Wilcoxon signed rank test was used without adjustments for multiple comparisons. P-values are as follows: Subject A: Syllable B, p=0.000; Syllable C, p=0.001; Syllable D, p=0.000; Syllable F, p=0.000; Syllable G, p=0.017; Subject B: Syllable B, p=0.353; Syllable C, p=0.001; Syllable E, p<=0.007; Subject C: Syllable D, f_{o1}, p=0.26; Syllable D, f_{o2}, p=0.025; Syllable E, p=0.001.
