# Supplementary material for: Wireless battery free fully implantable multimodal recording and neuromodulation tools for songbirds
Source: Nat Commun. 2021 Mar 30;12:1968. doi: 10.1038/s41467-021-22138-8 (PMC8009877; doi:10.1038/s41467-021-22138-8)
Supplement: Supplementary file 6 — Description of Additional Supplementary Files [file 41467_2021_22138_MOESM6_ESM.pdf]

Title: Supplementary Movie 1:
Description: Video demonstrating the capability to program three individual devices in the same magnetic field and trigger optogenetic stimulation with low latency.

Title: Supplementary Movie 2:
Description: Demonstration of multimodal operation and multi device addressing.
